# Effect of Nurse-Led Interventions in Reducing Post-Traumatic Stress Disorder Symptoms in Inpatients: A Systematic Review and Meta-Analysis

**DOI:** 10.1192/j.eurpsy.2025.275

**Published:** 2025-08-26

**Authors:** F. Oflaz, N. D. Kılınç

**Affiliations:** 1 Koç University, School of Nursing; 2 Koç University, Institute of Health Sciences; 3 İstanbul Arel University, Faculty of Health Sciences, Istanbul, Türkiye

## Abstract

**Introduction:**

Psychologically, traumatic incidents often involve physical injuries that threaten a person’s survival and sense of security. After a sudden health problem or other kind issues, hospital admission can be an experience that negatively affects psychological health and recovery, often including symptoms of post-traumatic stress disorder, anxiety, and depression^.^ Nurses who spend a longer time with patients can determine the initial changes in individuals and play a crucial role in the decision-making process.

**Objectives:**

This systematic review aimed to investigate the effect of nurse-led interventions in reducing post-traumatic stress disorder symptoms in inpatients.

**Methods:**

In this review, we investigated studies from Pubmed, Cochrane Library, Medline (OVID), Scopus, Web of Science, CINAHL, Dergipark and TR Dizin (a directory) databases that met the inclusion criteria. The methodological quality of the studies was assessed using the Cochrane Risk of Bias 1 tool. This study was performed based on the Guidelines of Systematic Reporting of Examination presented in the PRISMA checklist. The search protocol has been registered at the PROSPERO International Prospective Register of Systematic Reviews.

**Results:**

This systematic review included seven studies with a total sample size of 736 inpatients (intervention group: 350 participants; control group: 345 participants). The meta-analysis revealed that nurse-led interventions showed no statistically significant impact on post-traumatic stress disorder symptoms (SMD: -4,08, Z = .79, p = .43), anxiety (SMD: .28, Z = .18, p = .86) and depression (SMD: -.60, Z = .87, p = .38) in inpatients.

**Conclusions:**

Out of seven studies, five of them indicated that these interventions were not effective in reducing PTSD symptoms while in two studies, the interventions were found to be effective in alleviating PTSD symptoms. To effectively reduce or prevent post-traumatic stress symptoms in inpatients, it is recommended to integrate a comprehensive range of therapeutic, evidence-based practices into clinical settings, facilitated by a multidisciplinary team approach within a educative and collaborative working environment.

**Disclosure of Interest:**

None Declared

